# Low- and negative-pressure hydrocephalus in children, clinical features, treatment, prognosis and proposed mechanisms

**DOI:** 10.3389/fped.2025.1602767

**Published:** 2025-06-05

**Authors:** Binghong Chen, Yongxiang Zhang, Yajun Jiang, Wenzhong Mei, Yuanlong Zhang

**Affiliations:** ^1^ Department of Neurosurgery, Neurosurgery Research Institute, The First Affiliated Hospital, Fujian Medical University, Fuzhou, Fujian, China; ^2^ Department of Cadre Healthcare, The First Affiliated Hospital, Fujian Medical University, Fuzhou, Fujian, China; ^3^ Department of Neurosurgery, National Regional Medical Center, Binhai Campus of the First Affiliated Hospital, Fujian Medical University, Fuzhou, Fujian, China; ^4^ Fujian Provincial Institutes of Brain Disorders and Brain Sciences, First Affiliated Hospital, Fujian Medical University, Fuzhou, China

**Keywords:** low- and negative-pressure hydrocephalus, pediatrics, clinical features, mechanisms, Ommaya capsule

## Abstract

**Introduction:**

Low- and negative-pressure hydrocephalus (L&NPH) is not a rare clinical syndrome, often seen as a consequence of multiple cranial surgery, characterized by enlarged ventricles and paradoxically low intracranial pressure. L&NPH has also been reported in children, but only a few cases have been reported in the literature and understanding of the characteristics of L.NPH, treatment and prognosis in children is lacking.

**Methods:**

We performed a systematic analysis of 44 pediatric patients with L&NPH described in the literature and 4 patients treated at our institution.

**Results:**

The results indicated that the most common cause of L&NPH in children was craniotomy. More than half of children with L&NPH had surgery prior to onset of the disease, including cerebrospinal fluid (CSF) shunt surgery or CSF drainage. Conservative treatments include postural therapy, intermittent compression of the shunt pump to drain CSF, and in a small number of patients, the adjustment of the shunt pressure is effective, but the vast majority of patients (90.91%) ultimately require a shunt device repositioning and often require more than 2 days of external CSF drainage prior to surgery. After comprehensive treatment, 77.5% of pediatric patients with L&NPH recover to pre-existing hydrocephalus, while 22.5% have severe symptoms such as coma or vegetative state or even death, which are clearly associated with the progression of the underlying disease.

**Discussion:**

The pathophysiological mechanism may be the result of self-regulatory decompensation of CSF circulatory dynamics, brain relaxation due to excessive loss of interstitial fluid in brain tissue, and gradual increase in compliance.

## Introduction

Hydrocephalus is commonly considered a pathological accumulation of cerebrospinal fluid (CSF) in the intracranial space, described by Dr Harold L. Rekate in 2009 as a functional distension of the ventricular system due to insufficient passage of CSF from the point of formation in the brain to the point of absorption in the systemic circulation (1). The incidence of hydrocephalus in children is not uncommon, with around one case per 1,000 births in high-income countries, with neonatal hydrocephalus, congenital aqueduct stenosis, myelomeningocele and brain tumors being the main causes of morbidity (2–4). This may be higher in developing countries where neonatal infections the most common causative mechanism (5).

The term hydrocephalus usually refers to high pressure hydrocephalus, often accompanied by increased CSF pressure. However, another, more rarer, more insidious and highly misdiagnosed hydrocephalus with apparent differences in pathophysiology, clinical features, and treatment strategies, proposed as low-pressure hydrocephalus (LPH, 0< ICP <70 mmH_2_O) or negative-pressure hydrocephalus (NPH, ICP ≤0 mmH_2_O), is often overlooked (6). L&NPH is uncommon in clinical practice, but may lead to serious neurological impairment, even long-term bed rest, persistent coma and other poor prognosis (7).

L&NPH in children is one of the special types, which is obviously different from adult L&NPH in terms of etiology, treatment, and care methods, but existing studies are limited and consensus is lacking (8, 9). In this study, we retrospectively analyzed four L&NPH cases in children treated in our unit over the last few years, combined with a systematic literature review of the literature already published, to provide a summary of the clinical characteristics, pathogenesis and treatment of L&NPH in children, and to provide new insights into the mechanisms for the development of L&NPH.

## Material and methods

### Present patients enrolled

Clinical data from 4 patients in children with L&NPH were retrospectively analyzed at the Neurosurgery Department of the First Affiliated Hospital of Fujian Medical University from January 2017 to December 2023. The research was approved by the Ethics Committee of the First Affiliated Hospital of Fujian Medical University. All patients met the following criteria: (1) below 18 years of age; (2) prior shunt or external drain procedure in cerebrospinal fluid; (3) imaging confirming enlarged ventricles; (4) intracerebroventricular or lumbar puncture findings indicating intracranial pressure of below 70 mmH_2_O; and (5) improvement in clinical symptoms or imaging after shunt or external drainage procedures.

### Information sources, literature search and selection of study

Independent data on patients with L&NPH were obtained from published literature through a systematic review of all English-language publications published from 2003 to 2023. We searched both MEDLINE and EMBASE using the phrase “(“Low pressure hydrocephalus” OR “Negative pressure hydrocephalus”) AND [2003–2023]/py” respectively. Following deduplication of titles, two independent evaluators (B.H.C. and Y.L.Z.) reviewed the abstracts, and performed a full-text review and data extraction of relevant studies. Discrepancies at any stage of the review and data extraction were resolved by consensus between the evaluators under the guidance of the lead investigator (W.Z.M.). Other documents have been identified from the reference lists documents included.

The data extracted from the patient population age, gender, most likely cause of hydrocephalus (hemorrhage, neoplasm, trauma, congenital, infectious, or unknown), L&NPH-associated symptoms (headache, nausea, vomiting, cranial nerve paresis, or gait disturbance). In addition, we documented the patient's treatment history and prognosis. In order to be conservative in our reporting of occurrences, if a record is not specifically reported as being present, it was counted as an absent finding (NA).

### Statistical analysis

All statistical analyses were performed using IBM SPSS Statistics (version 28.0.0). Descriptive statistics on patient demographics, clinical factors, interventions and outcomes were presented as a percentage of the total number of patients with available records.

## Results

### Study enrolled

**Figure 1:**
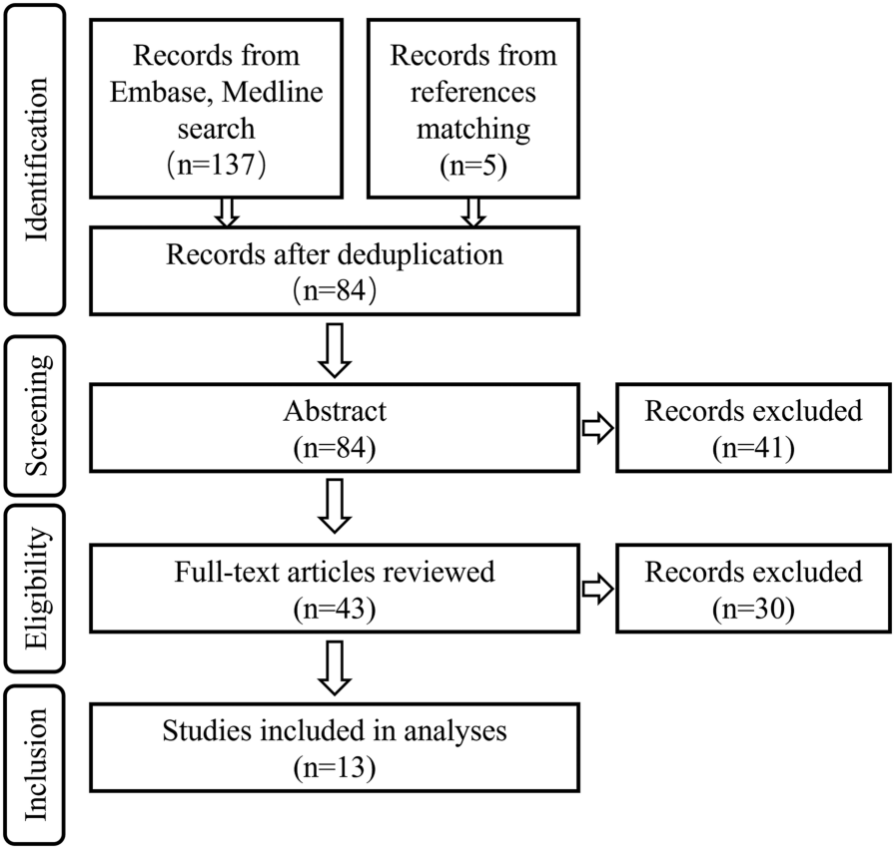
Flowchart of a systematic review of the existing literature (2003–2023) on patients with L&NPH.

In total, 142 articles were retrieved for this study, 84 from EMBASE and 58 from MEDLINE (5 from reference matching). Following deduplication and review of abstracts, 43 papers underwent full review, 30 of which were excluded for failure to meet inclusion criteria. Forty-four independent patients were identified from 13 papers (Figure 1). Overall, forty-eight patients were enrolled in this study, including 44 previously published cases and 4 unpublished cases from our institution.

### Characteristics of patients

Of the 48 patients, 30 were male and 18 female, with an average age of 8.5 (0.5–18) years for males and 6.19 (0.42–15) years for females. The predominant etiology of L&NPH in all patients was neoplasm, accounting for 70.83% of the total, followed by hemorrhage (14.58%) and traumatic brain injury (TBI) (8.33%). Other rare etiologies are mainly observed in congenital malformations such as Dandy-Walker malformations, complex craniosynostosis and stenosis of aqueduct, NF type I. The most common types of tumors are medulloblastoma and astrocytoma, accounting for 58.82% of all neoplasms. Other tumors that are relatively rare include atypical teratoid rhabdoid tumor, ependymoma, pineal tumor, choroid plexus papilloma and oligoastrocytoma. The second leading cause of L&NPH is cerebral hemorrhage, particularly post-hemorrhagic hydrocephalus of prematurity (PHHP). The most common symptoms in pediatric patients with L&NPH are fatigue, vomiting and headache, which are also the most common in adults. In pediatric patients, more specific symptoms including bradycardia, seizure and failure to thrive are observed (Table 1, Supplementary Table S1).

**Table 1:** Summary of patient demographics and clinical factors associated with L/NPH.

Variable	Male	Female	*P*-value
Sex	30 (62.5%)	18 (37.5%)	–
Age	8.50 ± 5.15	6.19 ± 5.27	0.816
Etiology of hydrocephalus			
Neoplasm	24	10	0.049
Astrocytoma	5	3	
Medulloblastoma	10	2	
Ependymoma	2	1	
Atypical teratoid rhabdoid tumor	2	3	
Pineal tumor	3	0	
Choroid plexus papilloma	1	1	
Oligoastrocytoma	1	0	
Traumatic brain injury	4	0	
Hemorrhage	1	6	
PHHP	1	4	
Others (Dandy–Walker malformation, complex craniosynostosis, Stenosis of aqueduct, NF type I)	1	2	–
Symptoms	*N*		
Drowsiness	20		–
Nausea/emesis/vomiting	19		–
Headache	16		–
Bradycardia	6		–
Seizure	6		–
Agitation	4		–
Altered consciousness	4		–
Dementia/akinetic mutism	3		–
Failure to thrive	2		–
Others (dizziness, dysconjugate gaze, decerebrate posturing, and slurred speech)	4		–
Unknown	2		–

### Previous treatment prior to L&NPH diagnosis and definitive treatment

Treatment before onset of L&NPH was documented in 46 patients. In addition to primary treatments, 15 (32.61%) patients were treated with ventriculoperitoneal shunt (VPS), 11 (32.61%) patients were treated with lumboperitoneal shunt (LPS), 3 (6.52%) patients were treated with external ventricular drainage (EVD), 2 (6.52%) patients were treated with endoscopic third ventriculostomy (ETV). Two of the three patients treated with EVD received concurrent VPS, and two of the patients treated with ETV received concurrent VPS. 19 (41.30%) patients did not receive any other treatment other than primary treatment and developed L&NPH spontaneously.

EVD subzero drainage, shunt adjustment, shunt replacement with special types such as anti-siphon shunts, anti-gravity shunts, etc. and conservative treatment are considered to be effective treatments for L&NPH (10, 11). Twenty-eight patients were documented to have received drainage, of which three drainage modalities were surgically placed ventricular drainage (57.14%), externalization of VPS (42.86%), and Ommaya puncture drainage (14.28%). Of the 28 patients documented, the average number of days of external CSF drainage was 41.89 days, with a minimum of 2 days and a maximum of 365 days. Endoscopic third ventriculostomy (ETV) is also a surgical intervention for L&NPH, but it seems to have limited effectiveness, three patients received ETV, but none of them has ever solved a problem using ETV alone, and all three patients required further external CSF drainage or shunt surgery (Table 2, Supplementary Table S1). Conservative treatment including maintain a semi-reclined position in bed, intermittently pressing the valve was also initiated to drain CSF, lumbar blood patch, downgrading a programmable valve setting, some patients are also able to improve their symptoms with conservative treatment.

**Table 2:** Treatment for 48 pediatric patients with L/NPH.

Variable	*N* (%)
Previous treatments prior to L/NPH diagnosis	
No. of records available	46
VPS, *n* (%)	15 (32.61%)
LPS, *n* (%)	11 (23.91%)
EVD, *n* (%)	3 (6.52%)
ETV, *n* (%)	2 (4.35%)
Spontaneous, *n* (%)	19 (41.30%)
Definitive treatment	
No. of records available	48
EVD inserted	
No. of records available	28
EVD, *n* (%)	12 (42.86%)
EVD time, days (min–max)	41.89 (2–365)
VPS externalized, *n* (%)	16 (57.14%)
Ommaya, *n* (%)	4 (14.29%)
VPS (or VAS)	44
VPS replacement or addition	40 (90.91%)
VPS revision or adjustment only	4 (9.09%)

### Outcomes

**Figure 2:**
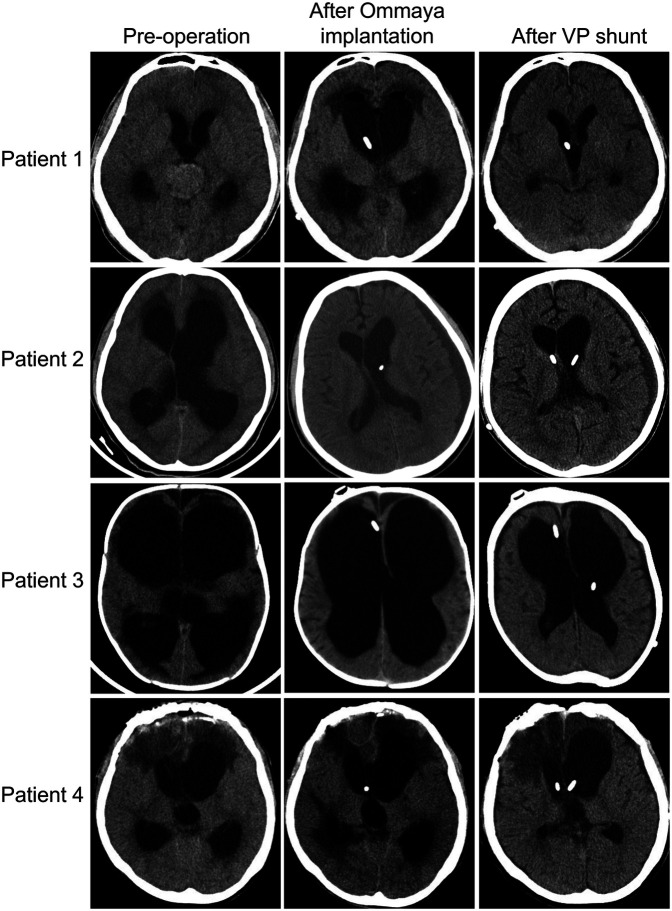
Cranial computed tomography images of four patients with L&NPH before hydrocephalus surgery, after Ommaya capsule implantation and after lateral VPS.

Out of the 40 patients with a definitive outcome reported in the literature, 31 patients (75%) recovered to baseline with the combination of treatment, 3 patients remained with severe symptoms (GOS <3), and 6 patients (13%) eventually died. Of course, the 31 patients who returned to baseline may also have had residual severe disease, but this has not been documented in the literature and severe disease is often associated with a primary illness. Among our four patients, all patients had a reduction in hydrocephalus on imaging after the treatment: first implantation of the Ommaya capsule and external drainage by capsule puncture, then switching to internal drainage (VPS) (Figure 2). One patient reverted to pre-L&NPH status after 21 days of treatment. Two patients also reverted to pre-L&NPH condition, but these two patients had more severe cognitive impairment due to primary disease before the onset of hydrocephalus, and the GOS was below 3 after L&NPH treatment. One patient eventually died due to the progression of intracranial infection (Table 3, Supplementary Table S1).

**Table 3:** Outcomes for 48 patients with L/NPH.

Outcome	Cases from literature	Our cases
No. of records available	40	4
Return to baseline, *n* (%)	31 (77.50%)	1 (25%)
Severe residual symptoms, *n* (%)	3 (7.50%)	2 (50%)
Died	6 (15.0%)	1 (25%)

## Discussion

### Summary of findings

The complexity of L&NPH in children is that it is a syndrome secondary to other conditions and more than half of the children received shunt surgery in addition to treatment for the primary disease. Shunt obstruction or shunt infection leads to multiple surgeries being performed as soon as the L&NPH is diagnosed. Shunt surgery and multiple procedures are risk factors for developing L&NPH (9, 12, 13). At the same time, the characteristics of children, such as heterogeneity of tumor etiology, heterogeneity of clinical signs, inability of children to express symptoms easily and childhood-related developmental problems, are a barrier to the identification of L&NPH in children.

Regarding primary disease, L&NPH in children is more likely to be secondary to brain tumors than in adults, which may be related to the spectrum of disease in both children and adults, rather than to the fact that post-operative patients with brain tumors are more likely to develop L&NPH (14). In our study, medulloblastoma and astrocytoma were the two major tumor etiologies of L&NPH in children, and these two tumors are also the most common in children (15). Diseases specific to children such as PHHP and developmental malformations, also contribute to L&NPH, which is rarely seen in adults. There is no significant difference in symptoms between adults and children, with headache, vomiting and decreased consciousness being the most common. However, children have relatively distinctive symptoms, such as bradycardia, seizure, and failure to thrive, which are less common in adults and require particular attention when treating pediatric patients.

Treatment of L&NPH in children follows the same principles as in adults. Treatment of L&NPH should be initiated after early diagnosis of hydrocephalus by direct manometry of CSF. Conservative treatment includes postural adjustment, lumbar puncture, manual compression of the shunt pump and adjustment of the pressure of the shunt. However, conservative treatment alone does not always solve the problem and further sequential EVD therapy and shunting is required. In this study, 24 patients received one or both of these two types of external CSF draining including externalization of EVD and VPS. Meanwhile, all four patients treated at our center were treated with external CSF draining from the reservoir Ommaya capsule. If the final decision to replace the VPS unit is made, special devices such as anti-gravity or anti-siphon valves are often required and the value must be set at a lower level. After sequential treatment with external CSF draining and shunting, the majority of patients can benefit, and in this study, 77.50% of the patients were able to return to the state before onset of L&NPH. But also 15% of patients die from L&NPH or primary disease progression, a slightly higher mortality rate than in adults. Unlike most of the methods reported in the literature, our four patients were treated with simple external drainage of CSF via Ommaya puncture, and the median time to external drainage (105 days) was significantly longer than in the literature (8). Although our approach ultimately resulted in significant symptomatic improvement in three patients, in increase the risk of intracranial infection, and one patient ultimately died from intracranial infection. In addition, due to the prolonged drainage time, further rehabilitation of the patient is delayed, affecting the patient's long-term quality of life.

### Proposed mechanisms of L&NPH

Several biomechanical hypotheses have been proposed to explain L&NPH, such as the viscoelastic model (16), which states that the CSF accumulation in the ventricles during the early stages of hydrocephalus increases the intraventricular pressure, which in turn leads to a progressive deformation of the viscoelastic body (brain tissue) and enlargement of the ventricles. The pressure in the brain chambers decreases when the ventricles expand to a certain extent, similar to the unloading of the pressure applied to the viscoelastic body. As the recovery of viscoelastic body deformation has a delayed effect, although the ventricles have been in a low pressure state at this time, the enlarged ventricles failed to return to normal synchronously and formed L&NPH. According to the hypothesis of the porous sponge model proposed by Hakim et al., brain tissue is like a porous sponge with viscoelastic properties (17). During the process of continuous expansion of the ventricle, the permeability of the ventricle wall increases due to mechanical stretching, cerebral ischemia and hypoxia, proliferation of periventricular glial cells. The brain parenchyma will continuously inhale more CSF from the ventricles, resulting in the formation of low pressure in the ventricles and L&NPH. Akins et al. (7) claimed that changes in L&NPH ventricular system pressure are similar in nature to changes in negative pressure in the pleural cavity during inhalation and exhalation.

Many researchers have found that L&NPH occurs in patients with CSF leakage after lumbar puncture (18), arachnoid cyst shunt (19), and skull base surgery (20) respectively, which suggests that the cause of L&NPH is the difference in pressure gradient between the ventricles and the subarachnoid space. The production of L&NPH is thought to be caused by a decrease in brain tissue compliance when the subarachnoid pressure is lower than the circulating pressure for various reasons. All of the biomechanical hypotheses above suggested that the change of brain tissue compliance is the underlying cause of L&NPH. However, it remains to be seen whether brain compliance increases or decreases in patients with L&NPH. In recent years, with the emergence of magnetic resonance elastography (MRE), the hardness of brain tissue can be directly measured by non-invasive methods *in vivo*, which provides the possibility to study the viscoelastic properties of the brain and explore the biomechanical hypothesis of L&NPH. Streitberger et al. (21) studied 20 NPH patients with multifrequency MRE and spring pot model and found that the brain tissue stiffness decreased by about 20% compared with the healthy control group. Olivero WC et al. (22) found that the hardness of brain parenchyma (1.62 kpa) in patients with L&NPH was only about half of normal (3.0 kpa) for the first time by MRE, which indicated that the brain tissue of patients with L&NPH was obviously soft and the compliance was significantly increased.

It is proposed that interstitial fluid produced by capillaries in the brain can enter the subarachnoid cavity through a narrow pore, compensating for loss of fluid balance on the surface of the brain when the CSF in the subarachnoid cavity is deduced. This is a biological mechanism of self-regulation and compensation of CSF circulation dynamics. Combined with this mechanism, we speculate that L&NPH is an extreme state of chronic hydrocephalus. The pathophysiological mechanism may be the result of CSF circulatory dynamics self-regulating decompensation, relaxation of the brain caused by excessive loss of interstitial fluid from the brain tissue, and gradual increase of compliance. If for any reason (e.g., inflammation, hemorrhage, CSF leakage, etc.) lead to subarachnoid adhesion and stenosis, and decreases in brain rhythm due to reduced cerebral arterial pulse on the brain surface, reducing the driving force of the CSF circulation and reducing the cerebral blood flow, leading to accumulation and pressure. Furthermore, the reduction of the volume of CSF or the leakage of CSF in the subarachnoid space causes a pressure gradient between the ventricles and the subarachnoid space, which resulted in the gradual expansion of the ventricle due to an increase in radial force. This inevitably triggers a biological mechanism of self-regulation and compensation of the CSF circulation dynamics by extruding the fluid from the brain tissues to the subarachnoid space (23). At this time, the brain tissue just like a sponge after being squeezed out of water, and the compliance increases. Although the pressure in the ventricles is decreasing, the soft-tissue parenchymal is not yet able to cope with the decreased intraventricular pressure because the brain is already more relaxed. The ventricles continue to expand, and the water in the brain parenchyma is further wrung out, so the vicious cycle continues. Until there is no more water in the brain tissue to be forced out, the brain parenchyma relaxes to the limit, the ventricular volume increases extremely, and the pressure in the ventricles drops to a minimum (lower than normal), leading to L&NPH.

This mechanism can also be demonstrated by another extreme state after hydrocephalus shunt, slit ventricle syndrome (SVS). The pressure gradient difference of SVS is just the opposite of that of L&NPH. Because of the excessive shunt, the pressure in the ventricles is lower than in the subarachnoid space, and the brain interstitial fluid was secreted towards the ventricle for compensation. However, due to the barrier of ependymal membrane in the ventricular wall (active transport), the interstitial fluid cannot readily be released into the ventricle and accumulates, leading to a significant increase in the water content of the brain tissue, increased stiffness and decreased compliance. L&NPH and SVS can therefore be considered as two extreme states of chronic hydrocephalus, resulting from deregulation of the CSF circulation due to various etiologies.

## Conclusions

L&NPH in children often goes secondary to the common childhood primary diseases, especially tumors, and to multiple procedures associated with the primary disease. The most common symptoms are lethargy, vomiting and headache. Early and accurate diagnosis, prompt treatment and sequential treatment with necessary external CSF draining and CSF shunting may improve the prognosis in most children. The pathophysiological mechanism may be the result of CSF circulatory dynamics self-regulating decompensation, the relaxation of the brain caused by excessive loss of interstitial fluid from the brain tissue, and gradual increase of compliance.

## Data Availability

The original contributions presented in the study are included in the article/Supplementary Material, further inquiries can be directed to the corresponding authors.
